# A combined approach of evolutionary game and system dynamics for user privacy protection in human intelligence interaction

**DOI:** 10.1038/s41598-025-98805-3

**Published:** 2025-04-21

**Authors:** Lan Yao, Qiyang Zhang, Shuai Deng

**Affiliations:** 1 School of Information Engineering, Hubei University of Chinese Medicine, Wuhan, People’s Republic of China; 2 School of Safety and Management Engineering, Hunan Institute of Technology, Hengyang, 421002 People’s Republic of China; 3 Hubei Shizhen Laboratary, Wuhan, People’s Republic of China

**Keywords:** Generative Artificial Intelligence (GenAI), Privacy protection, Three-party game model, Sensitivity analysis, Incentive mechanism, Communication and replication, Computational models

## Abstract

The rapid development of generative artificial intelligence (GenAI) has generated significant economic and social value, alongside risks to user privacy. For this purpose, this study investigates privacy protection in human-AI interaction by employing a combined approach of evolutionary game and system dynamics. A three-party game model was developed to analyze the interactive effects and evolution of privacy protection strategies among the government, GenAI company, and users. Sensitivity analysis through system dynamics simulations was conducted on four kinds of factors—government, company, users, and incentive mechanisms, to reveal how these factors influence the strategy choices of the three parties. The results suggest that the government’s reputation, subsidies, free-riding benefits, fines, rewards from GenAI company to users, and the cost–benefit considerations of all three parties are key factors affecting strategic decisions. Moderate fine and subsidy policies can effectively promote privacy protection, with subsidy policies proving to be more effective than penalty policies. This paper provides theoretical support and decision-making guidance for balancing technological development and privacy protection in human–AI interaction, contributing to the regulated and orderly development of Generative Artificial Intelligence.

## Introduction

In the rapid development of artificial intelligence technology, Generative Artificial Intelligence (GenAI), as an innovative technology, has shown great potential and application value in many fields. These fields include text generation, sound synthesis, and image creation^1,2^. However, the widespread use of GenAI also introduce significant privacy risks also introduce significant privacy risks^3^. Especially in the user data collection, storage, model training and other key links, there is a risk of privacy breaches^4^. Although some studies have focused on GenAI privacy issues, the existing literature lacks systematic solutions that balance technological innovation of GenAI with the protection of users’ privacy rights^5^. A critical challenge in the field of GenAI is how to improve the user experience by using the user-provided information while ensuring that user privacy is not violated^6^. Although some technologies such as differential privacy^7^ and federated learning^8^ have been proposed to reduce privacy risks, the actual effects and scope of application of these technologies are still controversial. Furthermore, their adaptability and effectiveness across different application scenarios have not been fully established^4^. In addition, the rapid development of GenAI technology also poses new challenges to existing privacy protection regulations. Given the potential privacy risks associated with GenAI technology, it is particularly important to conduct further research to develop effective privacy protection strategies^9^.

“Personal data” means any information relating to an identified or identifiable natural person (‘data subject’). An identifiable natural person is one who can be identified, directly or indirectly, in particular by reference to an identifier such as a name, an identification number, location data, an online identifier or to one or more factors specific to the physical, physiological, genetic, mental, economic, cultural or social identity of that natural person^10^. In the context of GenAI, privacy breaches are often more hidden and complex^11^.They may occur at various stages of the AI lifecycle, including data collection, storage, model training, inference, fine-tuning, and model deployment^4^.

Many studies have been performed on privacy protection issues. Previous researches on privacy protection are primarily conducted from the perspectives of technology and legal regulation. From a technological standpoint, significant progress has been made in the development of privacy protection techniques aimed at addressing user privacy violations. This is particularly evident in the domain of generative artificial intelligence (GenAI), where there have been many studies on privacy protection technology. Key techniques include differential privacy (DP)^12^, federated learning (FL)^13^, homomorphic encryption (HE)^14^, and secure multi-party computation (SMPC). Liu et al. offer a comprehensive review of privacy-protecting techniques in GenAI, specifically designed to mitigate risks such as model inversion, data breaches, and membership inference attacks. Large language models (LLMs), which are the core of GenAI, have the potential to expose sensitive information, thereby presenting significant privacy risks when processing and generating vast amounts of data. These risks include both passive privacy violations and active privacy attacks^3^. Yan et al. evaluate the efficacy and limitations of privacy protection mechanisms employed by LLMs at various stages, including pre-training, fine-tuning, and inference. Furthermore, they advocate for the integration of privacy protection policies throughout all business operations, emphasizing that "privacy by design" should be implemented by embedding privacy principles into product development and organizational processes^4^.

From the perspective of policy and legal regulation, Chaudhuri et al. found that government regulation plays a moderating role between users’ privacy concerns and online information disclosure, emphasizing the balance between the government’s protection of citizens’ privacy and data utilization^9^. The application of GenAI technology, especially in public administration and services, requires the government to formulate corresponding policies and regulations to ensure that the application of the technology does not violate the privacy rights of citizens. Ye et al. and Shi examined the specific privacy risks, data leakage risks, and threats to personal data posed by GenAI, particularly within the Chinese context. By analyzing the latest practices in privacy and personal data protection in China, these studies pointed out gaps in the existing legal framework and underscoring the urgent need for institutional reforms^15,16^. Through systematic reviews and in-depth interviews, Saura et al. identified the privacy risks involved in government use of AI and discussed the development of regulations to ethically design citizen data collection^17^. Wang and Wu raise the need for a harmonized legal framework to address the associated risks in the GenAI era, highlighting the importance of international cooperation, proactive legislation and the establishment of a dedicated regulatory body. This suggested that the legal dimension of GenAI is not limited to a single country, but also requires cooperation and harmonization of regulations across national borders^18^. Guo et al. combined technology acceptance and regulatory policy to highlight that different categories of regulatory policy tools reshape the perceived risks associated with the public’s use of technology and significantly affect the public’s perception of privacy^19^.

As the subject of privacy protection, user privacy decisions have attracted growing attention from researchers. Based on the privacy computing theory, users typically make disclosure or non-disclosure decisions after carefully evaluating the benefits and risks associated with revealing personal information^20^. Chaudhuri et al. study the antecedents of privacy concerns and their impact on consumers’ online information disclosure, and finds that privacy awareness, privacy experience, personality and cultural differences significantly affect privacy concerns, which in turn affect online information disclosure^9^. Additionally, consumer privacy decisions are malleable and can be influenced by commercial or governmental interests^21^. Users’ privacy disclosures may lead to leaks or threats. However, disclosing information to GenAI can also bring benefits. These include economic gains^22^, interpersonal relationship brought by shared information^23^, and improved service experiences. Beltran et al. provide a comprehensive framework for analyzing privacy in modern societies, emphasizing the importance of information privacy. It also outlines the structure of privacy values within citizen-government, employee-employer, and consumer-business relationships^24^. With the development of GenAI technology, public awareness of the privacy protection has increased. This has raised demands for transparency and trust from governments and companies. Consumer privacy decisions are a complex and dynamic process, influenced by environmental, psychological and social factors^6^.

To summarize, the current implementation of privacy protection in the field of GenAI mainly focuses on privacy protection technology and government policy supervision tools. However, there has been limited exploration into how the government influences users’ privacy disclosure behaviors. Furthermore, existing research remains within the scope of qualitative analysis and lacks a multi-stakeholder perspective. Notably, the roles of GenAI company, governments, and users—who collectively influence users’ privacy protection behaviors—are typically considered in isolation, rather than being integrated into a unified analytical framework. However, the strategic choices made by these three parties regarding privacy protection are interdependent, and their decisions evolve over time. While these parties share common interests, they also experience inherent conflicts. Given the bounded rationality of the government, GenAI company and users, the strategy selection process of these players is dynamic, as they continuously adjust their decisions by observing and matching their payoffs with those of others. Evolutionary game theory is particularly suited for capturing this dynamic process, as it models the evolution of strategies over time^25,26^. The evolutionary game theory approach has been widely applied in various scenarios, including IoT networks^27^, social networks^28^ and online shopping^29^. The convergence behavior of the system is affected by many factors such as the government’s reputation, incentive mechanisms, the cost for government regulation and rewards from company to users. Therefore, changes in specific factors can cause oscillations within the system, resulting in the emergence of a new equilibrium state^30^. Numerical simulation is used as the primary method to investigate these dynamics, as it enables detailed analysis of how different scenarios affect the course of the game^31^. A significant advantage of numerical simulation is its ability to analyze the dynamic behavior of complex phenomena over time. As a soft modeling approach, it is to describe the internal order underlying complex problems of the real world^32^. It emphasizes uncovering the internal structures and mechanisms behind complex real-world problems^33^, providing a powerful tool for studying the evolutionary dynamics of user privacy protection in human intelligence interaction.

By constructing a three-party game model involving the government, GenAI company and users, this study aims to analyze the strategic interactions among these stakeholders with respect to privacy protection, and to provide both theoretical insights and practical guidance for the formulation of effective privacy protection policies. Such contributions are crucial not only for safeguarding users’ privacy rights but also for fostering the sustainable development of GenAI technology. The key contributions of this paper are as follows: First, it builds on prior research by extending its focus to the current hot topic of GenAI. It integrates the three principal stakeholders in the GenAI privacy protection field into a cohesive framework, analyzing the impact of each party on the system’s evolution; Second, it introduces a government incentive and punishment mechanism to examine the strategic choices of these stakeholders through system dynamics, enabling the theoretical examination of a broader range of parameter scenarios. Third, it demonstrates the effectiveness of the model through numerical simulation and experimental verification, and analyze the influence of different parameters on privacy protection policies, providing specific guidance for practical privacy protection design in human intelligence interaction.

The rest of this paper is organized as follows. In “Assumptions and game model” section, dafter stating the research problem and basic assumptions, the multi-player evolutionary game model of privacy protection is developed. “Evolutionary analysis of the three parties” section analyzes the strategy stability of stakeholders. “System simulation and sensitivity analysis” section describes the system dynamics model structure and presents the system simulation results and discussion. “Conclusion and practical suggestions” section concludes main findings, proposes practical recommendations and discusses limitations and future research directions.

## Assumptions and game model

### Problem description of privacy protection in GenAI

**Fig. 1:**
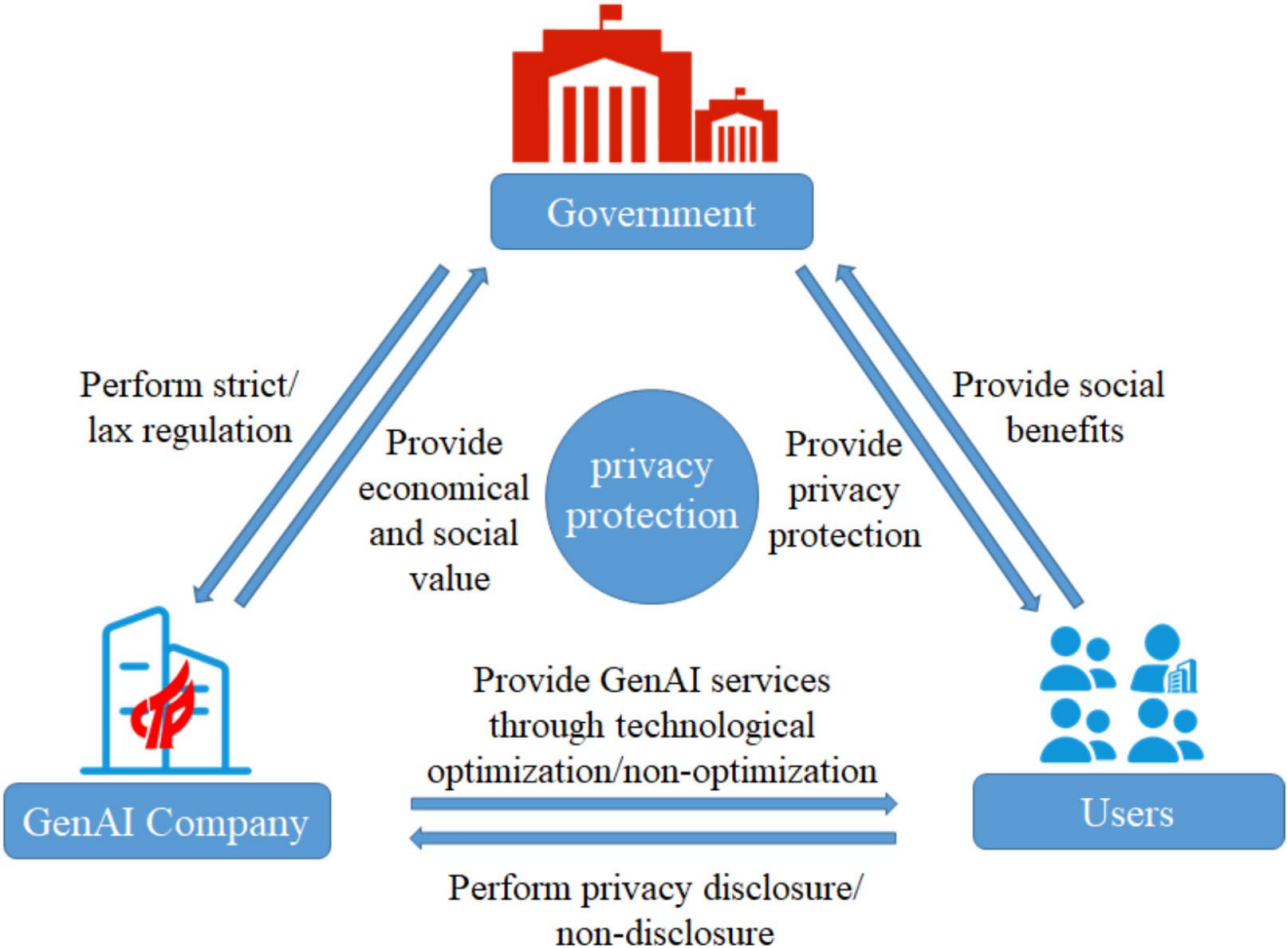
Three-party game model for privacy protection.

For the privacy protection problem of GenAI, this study develops a three-party game model for GenAI privacy protection as shown in Fig. 1. Among them, as the formulator and regulator of privacy protection policies, the government plays a crucial role in privacy protection in the field of GenAI. The government refers to government departments such as the Cyberspace Administration of China, the public security organs, the State Administration for Market Regulation, the Ministry of Industry and Information Technology, and provincial big data bureaus. By establishing comprehensive laws and regulations alongside implementing incentive and penalty mechanisms, the government not only drives advancements in social technology but also GenAI company to enhance users’ privacy protection through technological improvements. GenAI company refer to the company specializing in the development and application of generative artificial intelligence technologies, offering services through GenAI products such as ChatGPT, Wen Xiaoyan, Doubao, Kimi, Quark AI and DeepSeek.

Users, as the recipients of GenAI services, can decide whether to disclose their personal information when using these services. Non-disclosure refers to a strategy where users, concerned about potential privacy breaches, utilize GenAI products solely for task completion without sharing or sharing minimal personal information. In contrast, opting for disclosure implies that users are willing to provide personal data to access benefits such as discounts, rewards, enhanced service functionality, or personalized recommendations^22,23,34^. It is crucial for GenAI company to optimize privacy protection technologies. In the process of user interaction with GenAI, regardless of whether users choose to disclose personal information, potential risks of privacy breaches may arise during the collection, storage, and analysis of user data by GenAI company. To mitigate the risk of privacy breaches, GenAI company needs to take a series of technological optimization measures, including sensitive word filtering, data encryption, and anonymization processing, to protect user information from exploitation by malicious actors. However, these measures come with substantial additional costs, such as research and development expenses for security algorithms and privacy protection technologies, data management costs for tasks like annotation, encryption, and desensitization, as well as compliance costs related to legal requirements and auditing.

Based on the above game model, a decision-making interaction is formed among the government, GenAI company and users. Government regulatory strategies—such as policy formulation, financial support, incentive measures, and enforcement mechanisms, will directly influence the behavior of both GenAI company and users. Moreover, the regulatory intensity and policy orientation of the government play a crucial role in shaping the market environment, impacting the investment decisions of GenAI company^35^ and the trust of users^19^. The technological development and product optimization strategies of GenAI company will be influenced government policies and, in turn, influence user choices and market acceptance. The efforts of GenAI company in ensuring technological security, regulatory compliance, and user privacy protection not only enhance their brand image but may also attract government rewards or subsidies.

Users’ privacy disclosure strategy is critical to the growth of GenAI company. Active disclosure not only fosters improvements in GenAI company products and drives technological innovation^36,37^, but also affects the government’s regulatory strategies for the industry. Conversely, users’ privacy disclosure strategies for GenAI company will be affected by the companies’ technological optimization and government regulatory policies^38^.

Effectively coordinating the strategic choices of the government, GenAI company, and users to achieve a balance between technological development and privacy protection represents a complex and pressing challenge. This study constructs a three-party evolutionary game model considering the government’s reward and punishment mechanisms to analyze the interaction processes and evolutionary stable strategies of the three parties under different strategies. The findings offer theoretical guidance and practical recommendations for enhancing user privacy protection in the field of GenAI.

### Parameter description and assumptions

#### Parameter description

The main parameters mentioned in the research are descripted in Table 1.

**Table 1:** Parameters and meanings.

Parameters	Meaning
*C*_*A*_	The cost incurred by the government when opting for lax regulation
Δ*C*_*A*_	The additional cost borne by the government when implementing strict regulation
*S*	The reward provided by the government to a GenAI company for choosing optimization, such as tax incentives and research and development subsidies
*L*_*A*_	The loss of the government in the event of a risk occurrence
*R*_*BA*_	The benefits gained by the government from a GenAI company opting for optimization, including industrial development and economic growth
*M*	The free-riding benefits obtained by the government when GenAI company chooses optimization and users disclose privacy under lax regulation
*G*	The reputation value obtained by the government from implementing strict regulation, reflecting its public image and trustworthiness
*R*_*BH*_	The direct income generated by GenAI company by optimizing its algorithms to eliminate risky content, including improved user trust and enhanced brand image
*R*_*BL*_	The income of GenAI company when opting for non-optimization
*C*_*B*_	The cost paid by GenAI company
Δ*C*_*B*_	The additional cost borne by GenAI company during the optimization process
*L*_*B*_	The losses that GenAI company may encounter in the event of privacy breaches or other risk events, including economic losses and reputation damage
*F*_*AB*_	The fine imposed by the government on GenAI company in the event of a risk event (assuming that the probability of a risk event is 0 when both GenAI company and users are active, as in Assumption 6)
*p*_1_	The probability of a risk event when the GenAI company chooses non-optimization(*p*_1_ > *p*_2_)
*p*_2_	The probability of a risk event when the GenAI company chooses optimization
*R*_*CH*_	The income that users may gain by disclosing their privacy to the GenAI company
*R*_*CL*_	The income of users when choosing not to disclose privacy while using GenAI
*C*_*C*_	The costs paid by users when choosing not to disclose
Δ*C*_*C*_	The additional costs borne by users during the privacy disclosure process, such as time investment and concerns over privacy
*L*_*C*_	The losses that users may suffer in the event of privacy breaches or other risk events, including personal information breach and financial losses
*T*	The rewards offered by GenAI company that chooses optimization to incentivize user privacy disclosure, such as points, levels, VIP memberships, aimed at increasing user willingness to share information
*x*	The probability that the government opting for strict regulation
*y*	The probability that GenAI company opting for optimization
*z*	The probability that users opting for privacy disclosure

#### Basic assumptions

Based on the above problem description, this study proposes the following assumptions:

Assumption 1: The participants in the game are the government, the GenAI company, and users. All three are maximizing their own interests under the condition of bounded rationality, and the information among them is asymmetric.

Assumption 2: The government’s strategy set includes {strict regulation, lax regulation}. Under strict regulation, the government incurs additional costs compared to lax regulation. As reputation is a key concern, the government provides subsidies to the GenAI company that chooses optimization under strict regulation, with such subsidies accounted for in government expenditures. In the absence of risky events, such as privacy breaches, the government derives reputational benefits from strict regulation. Conversely, under lax regulation, if the GenAI company chooses optimization and users disclose their privacy, the government can reap certain free-riding benefits.

Assumption 3: The GenAI company’s strategy set includes {optimization, non-optimization}. When GenAI company chooses optimization, it incurs higher costs compared to non-optimization. These costs include, but are not limited to, expenses for training the AI core product, technical research and development of security algorithms and privacy protection technologies, data management efforts such as annotation, encryption, and desensitization, compliance costs for adhering to legal regulations and conducting audits, as well as emergency response costs for real-time monitoring and error remediation. At the same time, optimization enables the GenAI company to deliver superior products, garner better user feedback, attract new users, and generate higher revenue, i.e., *R*_*BH*_ > *R*_*BL*_. In addition, the GenAI company that chooses optimization contributes to societal and economic benefits for the government, driven by industrial development and economic growth. Conversely, if the GenAI company chooses non-optimization and the government enforces strict regulation, any occurrence of a risk event will result in the government imposing fines on the company as a punitive measure. These fines are accounted for as part of government revenue.

Assumption 4: The users’ strategy set includes {disclosure, non-disclosure}. When users choose to disclose privacy, they incur higher costs compared to non-disclosure, but they also have more income in the process of disclosure, which is reflected in obtaining better task support. For instance, users can improve their skills through disclosure, refine prompt accuracy, and achieve faster and more effective task completion with the assistance of GenAI. At the same time, since user disclosure facilitates the optimization of the GenAI company, the company will reward users, such as points, level rewards, and rights and interests.

Assumption 5: The probability that the government adopts strict regulation is denoted as *x*(0 ≤ *x* ≤ 1), while the probability of adopting lax regulation is (1−*x*); the probability that GenAI company chooses optimization is *y*(0 ≤ *y* ≤ 1), and the probability of choosing non-optimization is (1−*y*); the probability that users choose disclosure is* z*(0 ≤ *z* ≤ 1), and the probability of choosing non-disclosure is (1−*z*). The probability of a risk event occurring when the GenAI company chooses non-optimization is denoted as *p*_1_, and when it chooses optimization, the probability is *p*_2_. Given that continuous optimization allows the GenAI company to become more compliant, such as by avoiding more sensitive content, the probability of a risk event is lower when optimization is chosen, i.e., *p*_1>_*p*_2_.

Assumption 6: When a risk event occurs, all three parties, the government, GenAI company, and users, will suffer losses. However, when the GenAI company chooses optimization and users choose disclosure, it is assumed that the probability of privacy breaches and other risk events is so low to be ignored.

Based on the above problem description and assumptions, the payoff matrix of the three parties evolutionary game is obtained as Table 2.

**Table 2:** The payoff matrix.

	Government (strict regulation, lax regulation)	GenAI company (optimization, non-optimization)	Users (disclosure, non-disclosure)
(0,0,0)	*−p*_1_*L*_*A*_*−C*_*A*_	*−p*_1_*L*_*B*_*−C*_*B*_ + *R*_*BL*_	*−p*_1_*L*_*C*_*−C*_*C*_ + *R*_*CL*_
(0,0,1)	*−p*_1_*L*_*A*_*−C*_*A*_	*−p*_1_*L*_*B*_*−C*_*B*_ + *R*_*BL*_ + *R*_*CB*_	*−*(*C*_*C*_ + Δ*C*_*C*_) + *R*_*CH*_*−p*_1_*L*_*C*_
(0,1,0)	*−p*_2_*L*_*A*_*−C*_*A*_ + *R*_*BA*_	*−*(*C*_*B*_ + Δ*C*_*B*_) + *R*_*BH*_* −p*_2_*L*_*B*_	*R*_*BC*_*−p*_2_*L*_*C*_*−C*_*C*_ + *R*_*CL*_
(0,1,1)	*M−C*_*A*_ + *R*_*BA*_	*−*(*C*_*B*_ + Δ*C*_*B*_) + *R*_*BH*_ + *R*_*CB*_	*R*_*BC*_*−*(*C*_*C*_ + Δ*C*_*C*_) + *R*_*CH*_ + *T*
(1,0,0)	(1−*p*_1_)*G* + *p*_1_*F*_*AB*_*−p*_1_*L*_*A*_*−*(*C*_*A*_ + Δ*C*_*A*_)	*−p*_1_*F*_*AB*_*−p*_1_*L*_*B*_*−C*_*B*_ + *R*_*BL*_	*R*_*AC*_*−p*_1_*L*_*C*_*−C*_*C*_ + *R*_*CL*_
(1,0,1)	(1−*p*_1_)*G* + *p*_1_*F*_*AB*_*−p*_1_*L*_*A*_*−*(*C*_*A*_ + Δ*C*_*A*_)	*−p*_1_*F*_*AB*_*−p*_1_*L*_*B*_*−C*_*B*_ + *R*_*BL*_ + *R*_*CB*_	*R*_*AC*_*−*(*C*_*C*_ + Δ*C*_*C*_) + *R*_*CH*_*−p*_1_*L*_*C*_
(1,1,0)	(1−*p*_2_)*G* + *p*_2_*F*_*AB*_*−p*_2_*L*_*A*_ + *R*_*BA*_*−S−*(*C*_*A*_ + Δ*C*_*A*_)	*−p*_2_*F*_*AB*_*−*(*C*_*B*_ + Δ*C*_*B*_) + *R*_*BH*_ + *S−p*_2_*L*_*B*_	*R*_*AC*_ + *R*_*BC*_*−p*_2_*L*_*C*_*−C*_*C*_ + *R*_*CL*_
(1,1,1)	*G−*(*C*_*A*_ + Δ*C*_*A*_) + *R*_*BA*_*−S*	*−*(*C*_*B*_ + Δ*C*_*B*_) + *R*_*BH*_ + *S* + *R*_*CB*_	*R*_*AC*_ + *R*_*BC*_*−*(*C*_*C*_ + Δ*C*_*C*_) + *R*_*CH*_ + *T*

## Evolutionary analysis of the three parties

### Replicator equation

#### Government

Suppose the expected payoff of the government for strict regulation is *E*_*G*1_, the expected payoff for lax regulation is *E*_*G*2_, and the average payoff is *E*_*G*_.

Then the expected payoff *E*_G1_ for strict regulation is:

1 $$E_{G1} = G - \left( {C_{A} + \Delta C_{A} } \right) + y(R_{BA} - S) + \left( {{1} - y} \right)(p_{{1}} + zp_{{2}} ) \, (F_{AB} - G - L_{A} )$$

The expected payoff *E*_*G*2_ for lax regulation is:

2 $$E_{G2} = yzM - C_{A} + yR_{BA} - y\left( {{1} - z} \right)p_{{2}} L_{A} - \left( {{1} - y} \right)p_{{1}} L_{A}$$

The average expected payoff *E*_*G*_ is:

3 $$E_{G} = xE_{G1} + \left( {{1} - x} \right)E_{G2}$$

Therefore, the replicator equation for the government to choose strict regulation can be expressed as:

4 $$\begin{aligned} F_{G} & = dx/dt = x(E_{G1} - E_{G} ) = x\left( {{1} - x} \right)(E_{G1} - E_{G2} ) \hfill \\ & = x\left( {{1} - x} \right)[G - \Delta C_{A} - yS + \left( {1 - y} \right)\left( {p_{{1}} + zp_{{2}} } \right)\left( {F_{AB} - G} \right) - \left( {z - y} \right)p_{{2}} L_{A} - yzM] \hfill \\ \end{aligned}$$

The first order derivative *F*_*G*_^′^ of the government choosing strict regulation is:

5 $$F_{G}^{\prime} = dF_{G} /dx = \left( {{1} - {2}x} \right)[G - \Delta C_{A} - yS + \left( {1 - y} \right)(p_{{1}} + zp_{{2}} )\left( {F_{AB} - G} \right) - \left( {z - y} \right)p_{{2}} L_{A} - yzM]$$

Let *z*_*G*_ = [*G-*Δ*C*_*A*_-*yS* + (1*-y*)*p*_1_(*F*_*AB*_*-G*) + *yp*_2_*L*_*A*_]/[(1*-y*)*p*_2_(*G-F*_*AB*_) + *p*_2_*L*_*A*_ + *yM*], and by the stability of replicated dynamical equations there exists *z* such that:

1. When *z* = *z*_*G*_ and *F*_*G*_≡0, any value of *x* is stable, which indicates that the probability of the government adapting the strict regulation strategy does not affect its payoff.
2. When *z* ≠ *z*_*G*_, stability analysis of the differential equation indicates that the necessary conditions for the government to obtain higher payoffs by choosing strict regulation is *F*_*G*_ = 0 and *F*_*G*_^′^<0. Considering the value range of 0 < *z* < 1, there are the following two cases:

Case 1: When 0 < *z* < *z*_*G*_, there exists a constant *E*_*G*1_-*E*_*G*2_ > 0, so that *F*_*G*_'> 0 when *x* = 0, and *F*_*G*_'< 0 when *x* = 1. Therefore, *x* = 1 becomes the evolutionary asymptotic stability point (ESS), that is, the government stably adopts the strict regulation strategy.

Case 2: When *z*_*G*_ < *z* < 1, there exists a constant *E*_*G*1_-*E*_*G*2_ < 0, so that *F*_*G*_'< 0 when *x* = 0, and *F*_*G*_'> 0 when *x* = 1. Therefore, *x* = 0 becomes the evolutionary asymptotic stability point (ESS), that is, the government stably adopts the lax regulation strategy.

**Fig. 2:**
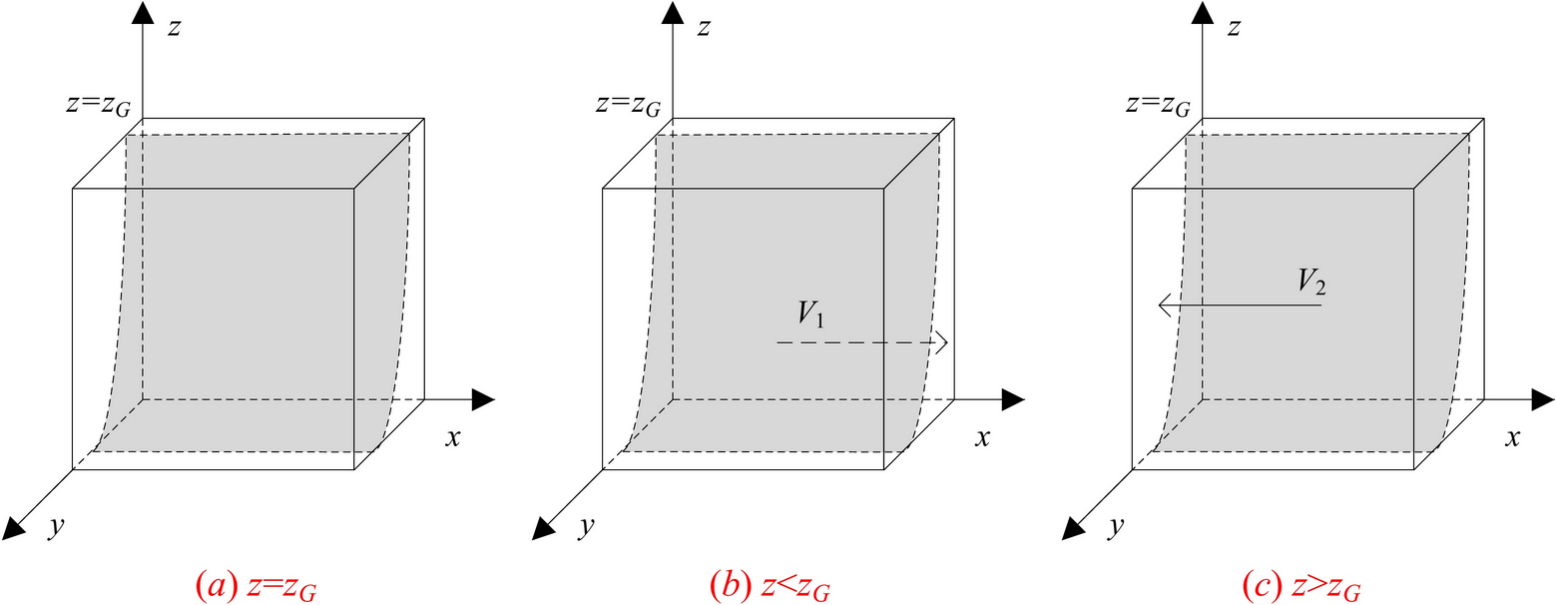
Phase diagram of government strategy evolution.

According to the replicated dynamic equation of the government, the evolutionary dynamic phase diagram is presented in Fig. 2.

**Inference 1**: When the initial state of the government’s strategy choice lies in the space* V*_1_, i.e., 0 < *z* < *z*_*G*_ and *x* = 1 is the equilibrium point, the government will adopt the strict regulation strategy. This is because more users prefer the non-disclosure strategy due to concerns about privacy breaches. The government’s strict regulation has a significant effect on the optimization phenomenon, thus obtaining a good social response. Therefore, the government’s decision-making tends to the strict regulation strategy.

**Inference 2**: When the initial state of the government’s strategy choice lies in the space *V*_2_, i.e., *z*_*G*_ < *z* < 1 and *x* = 0 is the equilibrium point, the government will adopt the lax regulation strategy. This is because when users use generative AI with peace of mind, the disclosure in the strategy choosing is at a relatively high level. As a result, the government’s strict regulation fails to significantly enhance optimization phenomenon of GenAI company, and the government will bear more cost losses. Consequently, the government’s decision-making tends to the lax regulation strategy.

#### GenAI company

Suppose the expected payoff of optimization for the GenAI company is *E*_*C*1_, the expected payoff of non-optimization is *E*_*C*2_, and the average expected payoff is *E*_*C*_.

Then the expected payoff of optimization *E*_*C*1_ is:

6 $$E_{C1} = - x\left( {{1} - z} \right)p_{{2}} F_{AB} - \left( {C_{B} + \Delta C_{B} } \right) + R_{BH} + xS + zR_{CB} - \left( {{1} - z} \right)p_{{2}} L_{B}$$

The expected payoff of non-optimization* E*_*C*2_ is:

7 $$E_{C2} = - xp_{{1}} F_{AB} - p_{{1}} L_{B} - C_{B} + R_{BL} + zR_{CB}$$

the average expected payoff *E*_*C*_ is:

8 $$E_{C} = xE_{{C{1}}} + (1 - x)E_{C2}$$

Therefore, the replicator equation for GenAI company to choose optimization in a dynamic environment can be expressed as:

9 $$\begin{aligned} F_{C} & = dy/dt = y(E_{C1} - E_{C} ) = y\left( {{1} - y} \right)(E_{C1} - E_{C2} ) \hfill \\ &= y\left( {{1} - y} \right)[ - x\left( {{1} - z} \right)p_{{2}} F_{AB} - \Delta C_{B} + R_{BH} + xS - \left( {{1} - z} \right)p_{{2}} L_{B} + xp_{{1}} F_{AB} + p_{{1}} L_{B} - R_{BL} ] \hfill \\ \end{aligned}$$

The first order derivative *F*_*C*_*'* of the GenAI company choosing optimization is:

10 $$F_{C}^{\prime} = dF_{C} /dy = ({1} - {2}y)[R_{BH} - x\left( {{1} - z} \right)p_{{2}} F_{AB} - \Delta C_{B} + xS - \left( {{1} - z} \right)p_{{2}} L_{B} + xp_{{1}} F_{AB} + p_{{1}} L_{B} - R_{BL} ]$$

Let *x*_*C*_ = [*R*_*BH*_-*R*_*BC*_ + *S*-Δ*C*_*B*_ + (*p*_1_-(1-*z*)*p*_2_)*C*_*B*_]/((1-*z*)*p*_2_-*p*_1_)* F*_*AB*_, and by the stability of replicated dynamical equations there exists *x* such that:

1. When *x* = *x*_*C*_ and *F*_*C*_≡0, any value of y is stable, which indicates that the probability of the GenAI company choosing the optimization strategy does not affect its payoff.
2. When *x* ≠ *x*_*C*_, stability analysis of the differential equation indicates that the necessary conditions for the GenAI company to obtain higher payoffs by choosing optimization is *F*_*C*_ = 0 and *F*_*C*_^′^<0. Considering the value range of 0 < *x* < 1, there are the following two cases:

When 0 < *x* < *x*_*C*_, there exists a constant *E*_*C*1_-*E*_*C*2_ < 0, so that *F*_*C*_'< 0 when *y* = 0, and *F*_*C*_'> 0 when *y* = 1. Therefore, *y* = 0 becomes the evolutionary asymptotic stability point (ESS), that is, the GenAI company stably adopts the non-optimization strategy.

When *x*_*C*_ < *x* < 1, there exists a constant *E*_*C*1_-*E*_*C*2_ > 0, so that *F*_*C*_'> 0 when *y* = 0, and *F*_*C*_'< 0 when *y* = 1. Therefore, *y* = 1 becomes the evolutionary asymptotic stability point (ESS), that is, the GenAI company stably adopts the optimization strategy.

**Fig. 3:**
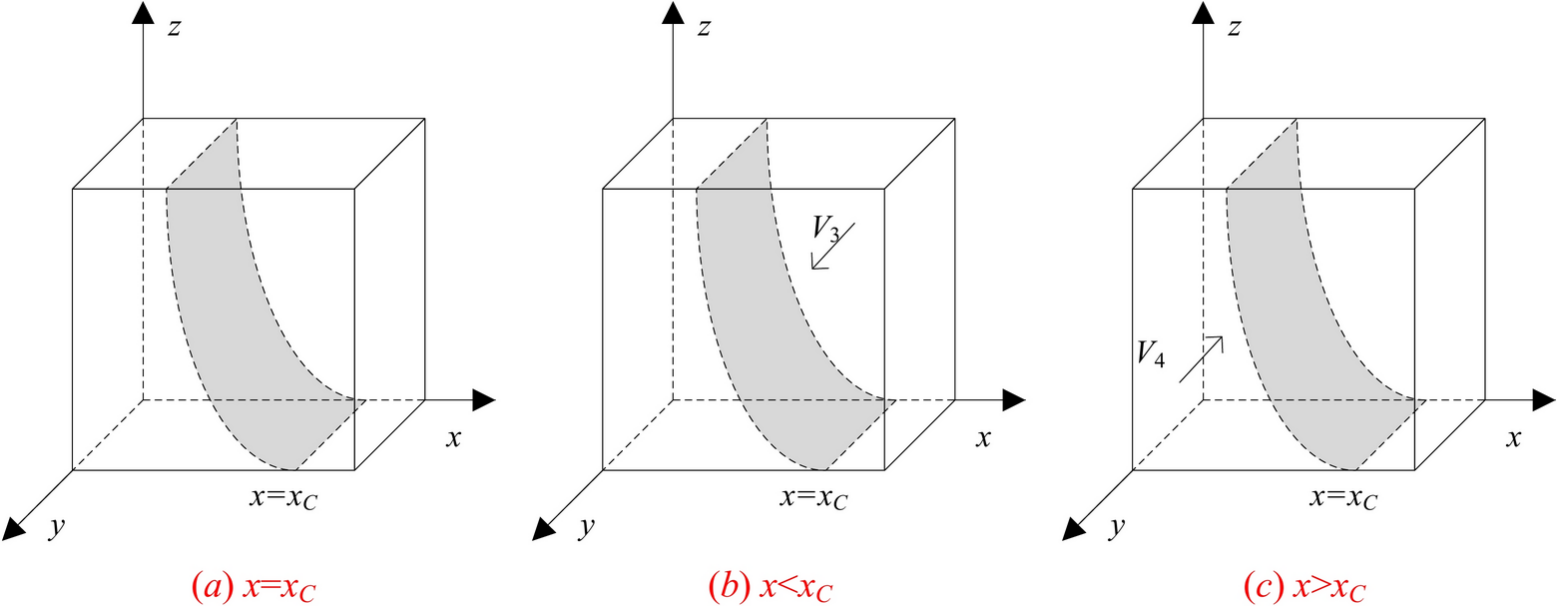
Phase diagram of GenAI company strategy evolution.

According to the replicated dynamic equation of GenAI company, the evolutionary dynamic phase diagram is presented in Fig. 3.

**Inference 3**: When the initial state of the GenAI company’s strategy choice lies in the space *V*_3_, i.e., *x*_*C*_ < *x* < 1 and *y* = 0 is the equilibrium point, the GenAI company will adopt the non-optimization strategy. This is because the disclosure in the user’s strategy selection is at a relatively high level, and the existing privacy protection means of the GenAI company can basically meet the user’s expectations for personal privacy protection.As a result, further optimization has minimal impact on users’ disclosure behavior. To minimize costs, the GenAI company’s decision-making tends to the non-optimization strategy.

**Inference 4**: When the initial state of the GenAI company’s strategy choice lies in the space *V*_4_, i.e., 0 < *x* < *x*_C_ and *y* = 1 is the equilibrium point, the GenAI company will adopt the optimization strategy. This is because the privacy disclosure in the user’s strategy selection is at a relatively low level, and the user’s privacy concerns are higher than the benefits brought by using GenAI products. In this case, optimization by the GenAI company significantly enhances user experience, resulting in improved user trust and better payoffs for the company. Therefore, the GenAI company’s decision-making tends to the optimization strategy.

#### User

Suppose the expected payoff of disclosure of users is *E*_*U*1_, the expected payoff of non-disclosure is *E*_*U*2_, and the average expected payoff is *E*_*U*_.

Then the expected payoff of disclosure *E*_*U*1_ is:

11 $$E_{U1} = - \left( {C_{C} + \Delta C_{C} } \right) + R_{CH} + y\left( {R_{BC} + T} \right) + xR_{AC} - \left( {{1} - y} \right)p_{{1}} L_{C}$$

The expected payoff of non-disclosure *E*_*U*2_ is:

12 $$E_{U2} = R_{CL} - C_{C} + y\left( {R_{BC} - p_{{2}} L_{C} } \right) + xR_{AC} - \left( {{1} - y} \right)p_{{1}} L_{C}$$

the average expected payoff *E*_*U*_ is:

13 $$E_{U} = zE_{U1} + \left( {{1} - z} \right)E_{U2}$$

Therefore, the replicator equation for users to choose disclosure can be expressed as:

14 $$F_{U} = z\left( {{1} - z} \right)\left[ {R_{CH} - \Delta C_{C} + y\left( {p_{{2}} L_{C} + T} \right) - R_{CL} } \right]$$

The first order derivative *F*_*U*_′ of users choosing disclosure is:

15 $$F_{U}^{\prime} = \left( {{1} - {2}z} \right)\left[ {R_{CH} - \Delta C_{C} + y\left( {p_{{2}} L_{C} + T} \right) - R_{CL} } \right]$$

Let *y*_*U*_ = (*R*_*CL*_ + Δ*C*_*C*_-*R*_*CH*_)_*/*_(*p*_2_*L*_*C*_ + *T*), and by the stability of replicated dynamical equations there exists *z* such that:

1. When *y* = *y*_*U*_ and *F*_*U*_≡0, any value of *z* is stable, which indicates that the probability of users choosing the disclosure strategy does not affect its payoff.
2. When *y* ≠ *y*_*U*_, stability analysis of the differential equation indicates that the necessary conditions for users to obtain higher payoffs by choosing disclosure is* F*_*U*_ = 0 and* F*_*U*_^′^<0. Considering the value range of 0 < *z* < 1, there are the following two cases:

When 0 < *y* < *y*_*U*_, there exists a constant *E*_*U*1_-*E*_*U*2_ < 0, so that *F*_*U*_'< 0 when *z* = 0, and *F*_*U*_'> 0 when *z* = 1. Therefore, *z* = 0 becomes the evolutionary asymptotic stability point (ESS), that is, the users stably adopt the non-disclosure strategy.

When *y*_*U*_ < *y* < 1, there exists a constant *E*_*U*1_-*E*_*U*2_ > 0, so that *F*_*U*_'> 0 when *z* = 0, and *F*_*U*_'< 0 when *z* = 1. Therefore,* z* = 1 becomes the evolutionary asymptotic stability point (ESS), that is, the users stably adopt the disclosure strategy.

**Fig.4:**
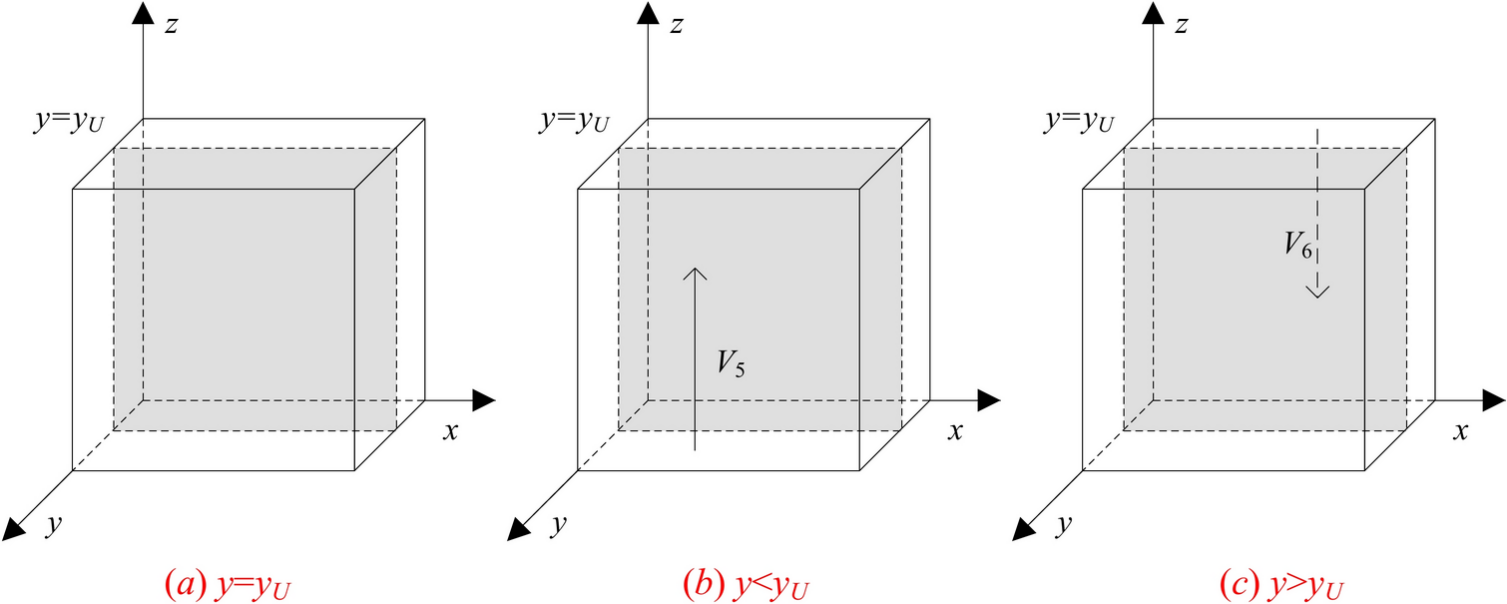
Phase diagram of users strategy evolution.

According to the replicated dynamic equation of users, the evolutionary dynamic phase diagram is presented in Fig. 4.

**Inference 5**: When the initial state of the users’ strategy choice lies in the space *V*_5_, i.e., *y*_*U*_ < *y* < 1 and *z* = 1 is the equilibrium point, the users will adopt the disclosure strategy. This is because the optimization in the GenAI company’s strategy selection is at a relatively high level, and the users’ disclosure behavior can be well protected in terms of privacy. In order to obtain a better GenAI experience, the users’ decision-making tends to the disclosure strategy.

**Inference 6**: When the initial state of the users’ strategy choice lies in the space *V*_6_, i.e., 0 < *y* < *y*_*U*_ and *z* = 0 is the equilibrium point, the users will adopt the non-disclosure strategy. This is because the optimization in the GenAI company’s strategy selection is at a relatively low level, so that the users’ disclosure behavior has a high risk of privacy breaches. Therefore, the users’ decision-making tends to the non-disclosure strategy.

### Evolutionary stability analysis

Evolutionary Stable Strategy (ESS) refers to a condition in which each game participant strives to maximize its own interests by continuously learning, imitating and adjusting its strategies, and ultimately achieving the evolutionary stable state of the system.

By simultaneously considering the three replicator dynamic equations, when *F*_*G*_ = 0, *F*_*C*_ = 0 and *F*_*U*_ = 0, it is a necessary condition for extreme values, which can generate equilibrium points in the evolutionary game dynamics. For the three parties, namely the government, GenAI company, and users, it is only necessary to discuss the asymptotic stability of eight points: *E*_0_(0,0,0), *E*_1_(0,0,1), *E*_2_(0,1,0), *E*_3_(0,1,1), *E*_4_(1,0,0), *E*_5_(1,0,1), *E*_6_(1,1,0) and* E*_7_(1,1,1). The asymptotic stability of the equilibrium points is determined by the Lyapunov’s discriminant method. Therefore, the Jacobian matrix and its eigenvalues are computed. The first-order partial derivatives of *F*_*G*_, *F*_*C*_ and *F*_*U*_ with respect to *x*, *y*, and *z* are calculated, and the Jacobian matrix of this system is as follows:

16 $$J = \left( {\begin{array}{*{20}c} {\partial F_{G} /\partial x} & {\partial F_{G} /\partial y} & {\partial F_{G} /\partial z} \\ {\partial F_{C} /\partial x} & {\partial F_{C} /\partial y} & {\partial F_{C} /\partial z} \\ {\partial F_{U} /\partial x} & {\partial F_{U} /\partial y} & {\partial F_{U} /\partial z} \\ \end{array} } \right)$$

where *∂F*_*G*_*/∂x* = (1*-*2*x*)[*G-*Δ*C*_*A*_*-yS* + (1*-y*)(*p*_1_ + *zp*_2_)(*F*_*AB*_*-G*)*-*(*z-y*)*p*_2_*L*_*A*_*-yzM*], *∂F*_*G*_*/∂y* = *x*(1*-x*)[*-S-*(*p*_1_ + *zp*_2_)(*F*_*AB*_*-G*) + *p*_2_*L*_*A*_*-zM*], *∂F*_*G*_*/∂z* = *x*(1*-x*)[(1*-y*)*p*_2_(*F*_*AB*_*-G*)*-p*_2_*L*_*A*_*-yM*], *∂F*_*C*_*/∂x* = *y*(1-*y*)[*-*(1-*z*)*p*_2_*F*_*AB*_ + *S* + *p*_1_*F*_*AB*_], *∂F*_*C*_*/∂y* = (1–2*y*)[*-x*(1-*z*)*p*_2_*F*_*AB*_*-*Δ*C*_*B*_ + *R*_*BH*_ + *xS-*(1-*z*)*p*_2_*L*_*B*_ + *xp*_1_*F*_*AB*_ + *p*_1_*L*_*B*_*-R*_*BL*_], *∂F*_*C*_*/∂z* = *y*(1-*y*)[*xp*_2_*F*_*AB*_ + *p*_2_*L*_*B*_], *∂F*_*U*_*/∂x* = 0, *∂F*_*U*_*/∂y* = *z*(1-*z*)(*p*_2_*L*_*C*_ + *T*)**,** *∂F*_*U*_*/∂z* = (1–2*z*)[*R*_*CH*_*-*Δ*C*_*C*_ + *y*(*p*_2_*L*_*C*_ + *T*)-*R*_*CL*_].

According to Lyapunov’s discriminant method, the condition for an equilibrium point to be evolutionarily stable (ESS) is that all eigenvalues* λ* of its corresponding Jacobian matrix must be less than 0. The stability analysis of each equilibrium point is summarized in Table 3.

**Table 3:** Equilibrium point stability analysis.

Equilibrium points	Government	Company	User	Stability
*E*_0_ = (0,0,0)	(1-*p*_1_)*G* + *p*_1_*F*_*AB*_-Δ*C*_*A*_	*R*_*BH*_-*R*_*BL*_-Δ*C*_*B*_ + (*p*_1_-*p*_2_)*L*_*B*_	-Δ*C*_*C*_ + *R*_*CH*_-*R*_*CL*_	Uncertainty
*E*_1_ = (0,0,1)	(1-*p*_1_)*G* + *p*_1_*F*_*AB*_-Δ*C*_*A*_	*R*_*BH*_-*R*_*BL*_-Δ*C*_*B*_ + *p*_1_*L*_*B*_	-[-Δ*C*_*C*_ + *R*_*CH*_-*R*_*CL*_]	Uncertainty
*E*_2_ = (0,1,0)	(1-*p*_2_)*G* + *p*_2_*F*_*AB*_-Δ*C*_*A*_	-[*R*_*BH*_-*R*_*BL*_-Δ*C*_*B*_ + (*p*_1_-*p*_2_)*L*_*B*_]	-Δ*C*_*C*_ + *R*_*CH*_ + *T* + *p*_2_Lc-*R*_*CL*_	Unstable
*E*_3_ = (0,1,1)	*G-M-S*-Δ*C*_*A*_	-[*R*_*BH*_-*R*_*BL*_-Δ*C*_*B*_ + *p*_1_*L*_*B*_]	-[-Δ*C*_*C*_ + *R*_*CH*_ + *T* + *p*_2_Lc-*R*_*CL*_]	Uncertainty
*E*_4_ = (1,0,0)	-[(1-*p*_1_)*G* + *p*_1_*F*_*AB*_-Δ*C*_*A*_]	*R*_*BH*_-*R*_*BL*_-Δ*C*_*B*_ + *S* + (*p*_1_-*p*_2_)(*L*_*B*_ + *F*_*AB*_)	-Δ*C*_*C*_ + *R*_*CH*_-*R*_*CL*_	Uncertainty
*E*_5_ = (1,0,1)	-[(1-*p*_1_)*G* + *p*_1_*F*_*AB*_-Δ*C*_*A*_]	*R*_*BH*_-*R*_*BL*_-Δ*C*_*B*_ + *S* + *p*_1_(*L*_*B*_ + *F*_*AB*_)	-[-Δ*C*_*C*_ + *R*_*CH*_-*R*_*CL*_]	Uncertainty
*E*_6_ = (1,1,0)	-[(1-*p*_2_)*G* + *p*_2_*F*_*AB*_-Δ*C*_*A*_]	-[*R*_*BH*_-*R*_*BL*_-Δ*C*_*B*_ + *S* + (*p*_1_-*p*_2_)(*L*_*B*_ + *F*_*AB*_)]	-Δ*C*_*C*_ + *R*_*CH*_ + *T*-*p*_2_Lc-*R*_*CL*_	Uncertainty
*E*_7_ = (1,1,1)	-[*G-M-S*-Δ*C*_*A*_]	-[*R*_*BH*_-*R*_*BL*_-Δ*C*_*B*_ + *S* + *p*_1_(*L*_*B*_ + *F*_*AB*_)]	-[-Δ*C*_*C*_ + *R*_*CH*_ + *T*-*p*_2_Lc-*R*_*CL*_]	Uncertainty

## System simulation and sensitivity analysis

### System dynamics model

**Fig. 5:**
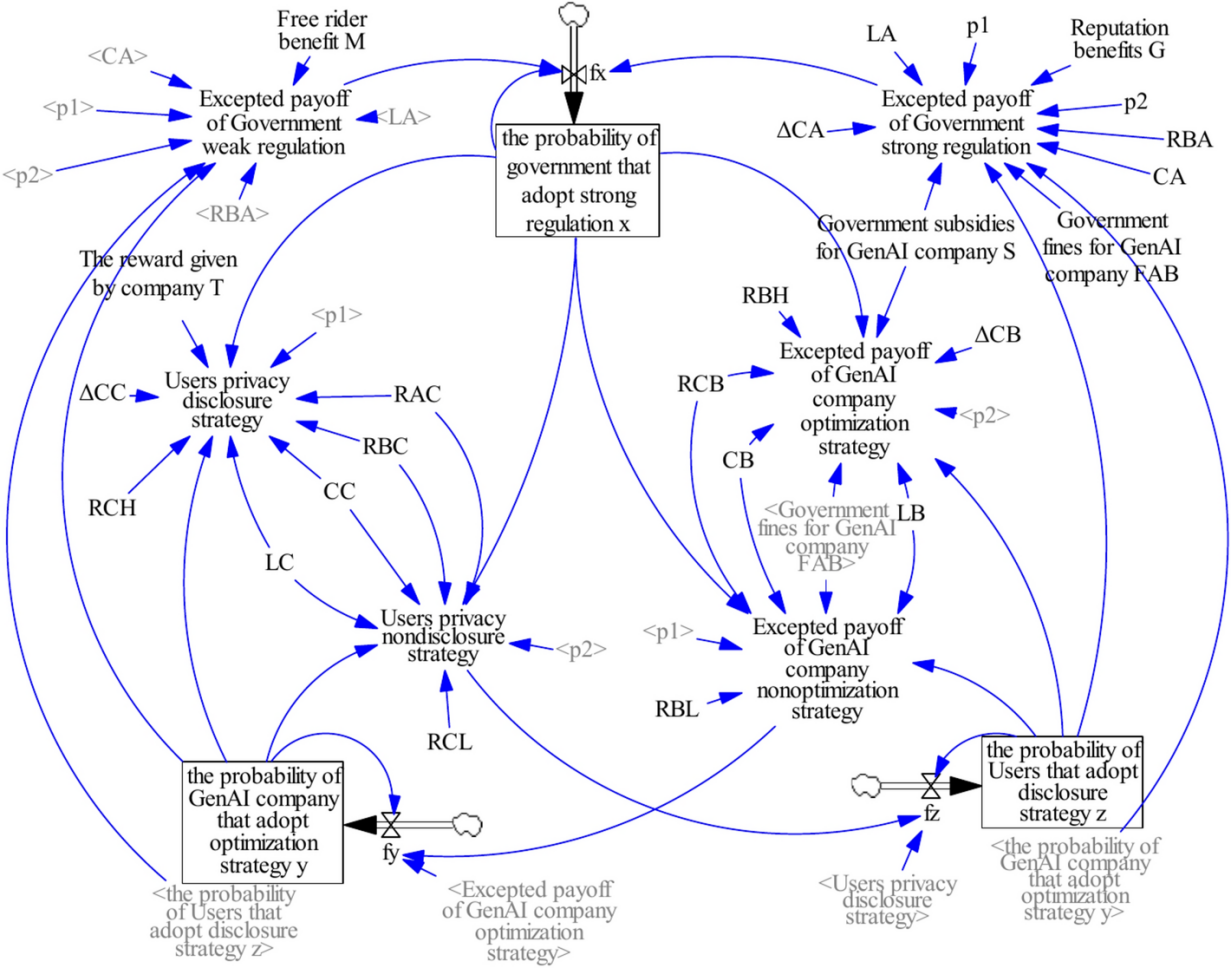
System dynamics diagram.

In order to examine the impact of the main parameters on the three-party game model, the replicator dynamic equations in the evolutionary game theory (EGT) model are integrated with the state change equations of the system dynamics (SD) model. The SD model is constructed using Vensim PLE.7.5b software, based on Eqs. (1) to (15). As shown in Fig. 5, the three state variables in the model represent the probability of each participant’s strategy choice in the three-party game (*x*, *y*,* z*), the three state rates are defined by the replicator dynamic equations (*fx*, *fy*, *fz*), and it also includes 6 auxiliary variables and 23 external parameters considered in this study. The state variables correspond to the probability of each participant’s strategy choice in the three-party game(*x*, *y*,* z*), while the rate variables include the change rate of participants’ strategy choices (*fx*, *fy*, *fz*).

Although this study is limited by the absence of real data which can be analyzed to get accurate results, the main purpose is to analyze the longitudinal evolutionary behavior of players^39^. The parameters are detailed in Table 1. The main purpose of this study is to analyze the evolution process of participants’ strategy choices over time. Therefore, the parameter values are adjusted to satisfy the conditions of the seven equilibrium points presented in Table 3 in the simulation analysis. Nevertheless, we set the parameter values as close to reality as possible to ensure rationality. The values of parameters are based on the data of survey reports, industry reports and government documents and experts’ opinions. The details are as follows: (1) the probability of a risk event parameters: The values of the probability (*p*_1_) was set based on the *APP Personal Information Leakage Investigation Report* released by China Consumers Association in 2018^40^. In terms of the main ways of information leakage, the survey found that criminals stole personal information through Trojan viruses and phishing websites, and operators collected unnecessary personal information accounted for 34.4 percent and 26.2 percent respectively. So we set *p*_1_ to be 0.3. According to *2025 State of Privacy report*by Information System Audit and Control Association (ISACA), most companies have adopted the principle of "privacy by design" when developing new applications, but it is not entirely safe from leaks^41^. So we set *p*_2_ to a lower value of 0.1. (2) the reward parameters: The *Enterprise Income Tax Law of the People’s Republic of China* stipulates that high-tech enterprises that need key support from the state shall be taxed at a 15% tax rate^42^, which means that the enterprise income tax rate will be reduced from 25 to 15%. Some regions provide subsidies for research and development (R&D) investments in high-tech enterprises. For example, the Rongchang District People’s Government of Chongqing offers a maximum subsidy of 300,000 yuan to enterprises recognized as national high-tech enterprises for the first time^43^. Taking these factors into comprehensive consideration, we set *S* as 10% of *C*_*B*_. (3) The costs and losses parameters: IBM’s *Cost of a Data Breach Report 2024* analyzed real data breach incidents across 604 global organizations between March 2023 and February 2024, revealing an average global breach cost of $4.88 million in 2024. 70% of organizations experienced significant or highly severe business disruptions due to breaches, with deeper levels of disruption correlating to higher average breach costs. The report further highlighted that enterprises extensively adopting security AI and automation technologies detected and contained breaches 98 days faster than those without such implementations^44^. So we set *L*_*B*_ as 20% of *C*_*B*_。Other parameters, such as free-riding benefits (*M*), user losses (*L*_*C*_), and user rewards from GenAI company (*T*), are challenging to determine empirically. To ensure the rationality of parameter values, consultations were conducted with GenAI industry practitioners and information management staff from the Wuhan Municipal Bureau of Data (https://home.wuhan.gov.cn/) and the Hubei Provincial Development and Reform Commission (https://fgw.hubei.gov.cn/). The initial values of the specific parameters are shown in Table 4.

**Table 4:** Parameter settings.

Parameter name	Parameter value						
	*E*_0_	*E*_1_	*E*_3_	*E*_4_	*E*_5_	*E*_6_	*E*_7_
*p*_1_	0.3	0.3	0.3	0.3	0.3	0.3	0.3
*p*_2_	0.1	0.1	0.1	0.1	0.1	0.1	0.1
*C*_*A*_	2	2	2	2	2	2	2
*C*_*B*_	5	5	5	5	5	5	5
*C*_*C*_	1	1	1	1	1	1	1
Δ*C*_*A*_	1	1	1	1	1	1	1
Δ*C*_*B*_	3	3	**1**	3	3	**1**	1
Δ*C*_*C*_	0.5	0.5	0.5	0.5	0.5	0.5	0.5
*L*_*A*_	1	1	1	1	1	1	1
*L*_*B*_	1	1	1	1	1	1	1
*L*_*C*_	1	1	1	1	1	1	1
*M*	2	2	2	2	2	2	2
*F*_*AB*_	0.5	0.5	0.5	0.5	0.5	0.5	0.5
*R*_*BA*_	1	1	1	1	1	1	1
*S*	0.5	0.5	0.5	0.5	0.5	0.5	0.5
*R*_*BH*_	2	2	2	2	2	2	2
*R*_*BL*_	1	1	1	1	1	1	1
*R*_*CH*_	1.2	**1.8**	1.2	1.2	**1.8**	1.2	1.2
*R*_*CL*_	1	1	1	1	1	1	1
*R*_*BC*_	1	1	1	1	1	1	1
*R*_*AC*_	1	1	1	1	1	1	1
*T*	0.1	0.1	**0.3**	0.1	0.1	0.1	**0.3**
*G*	1	1	1	**4**	**4**	**4**	**4**

### Equilibrium point stability analysis

**Fig. 6:**
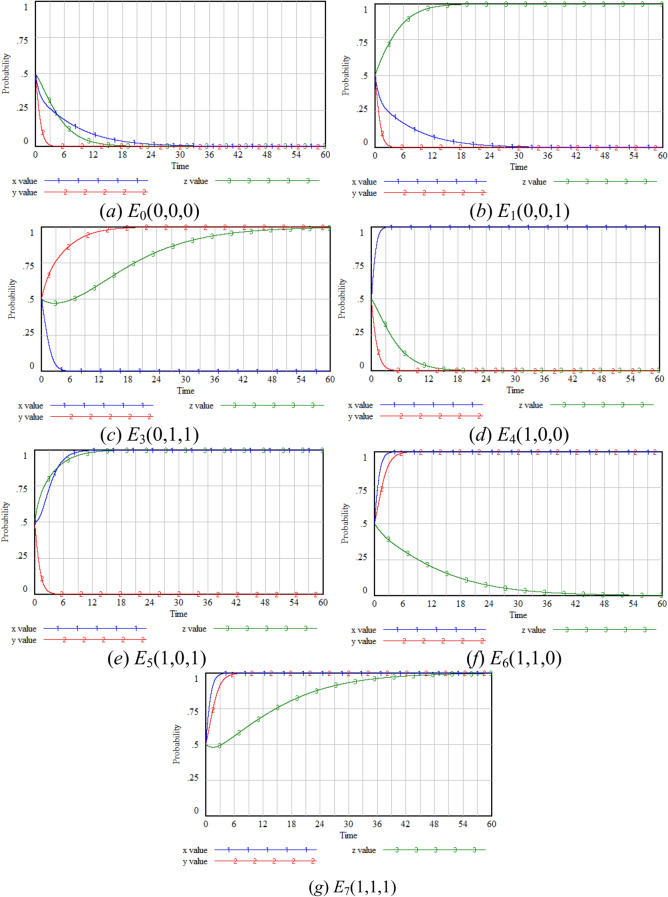
Stability test of equilibrium points.

To determine the sensitivity analysis scenarios and the selection of main parameters for each participant, the initial probabilities (*x*_0_, *y*_0_, *z*_0_) = (0.5,0.5,0.5) are chosen. Based on the system dynamics model and the associated parameter settings, simulations are conducted on the seven possible equilibrium points, with the results presented in Fig. 6.

Based on Table 3 and Fig. 6, the sensitivity analysis is divided into four groups: government sensitivity analysis, GenAI company sensitivity analysis, users’ sensitivity analysis, and incentive mechanism sensitivity analysis. Among them, the government, GenAI company, and users sensitivity analysis primarily examine the willingness of each participant in improving privacy protection through optimization in GenAI. Specifically, the parameters *G*, *M* and Δ*C*_*A*_ are analyzed for the government under the *E*_0_(0,0,0) scenario, the parameters *R*_*BH*_, *L*_*B*_ and Δ*C*_*B*_ are analyzed for the GenAI company, and the parameters *R*_*CH*_, *L*_*C*_ and Δ*C*_*C*_ are analyzed for the users. *F*_*AB*_ and *S* are the influence variables of the government on the GenAI company. Therefore, *F*_*AB*_ is analyzed under the *E*_0_(0,0,0) scenario, *S* is analyzed under the *E*_4_(1,0,0) scenario, and the change of the *T* value is analyzed under the *E*_6_(1,1,0) scenario to illustrate the impact of the rewards given by the GenAI company that chooses the optimization strategy to the users on the entire system.

### Government sensitivity analysis

With the aim of analyzing the impact of variables such as reputation value (*G*), free-riding benefits (i.e., *M*), and strict regulation costs (Δ*C*_*A*_) on the probability (*x*) of the government adopting the strict regulation strategy when addressing the privacy protection issues in the use of GenAI. We set six values ranging from small to large for each of *G*, *M*, and Δ*C*_*A*_, while the other variables remain constant at initial values.

**Fig. 7:**
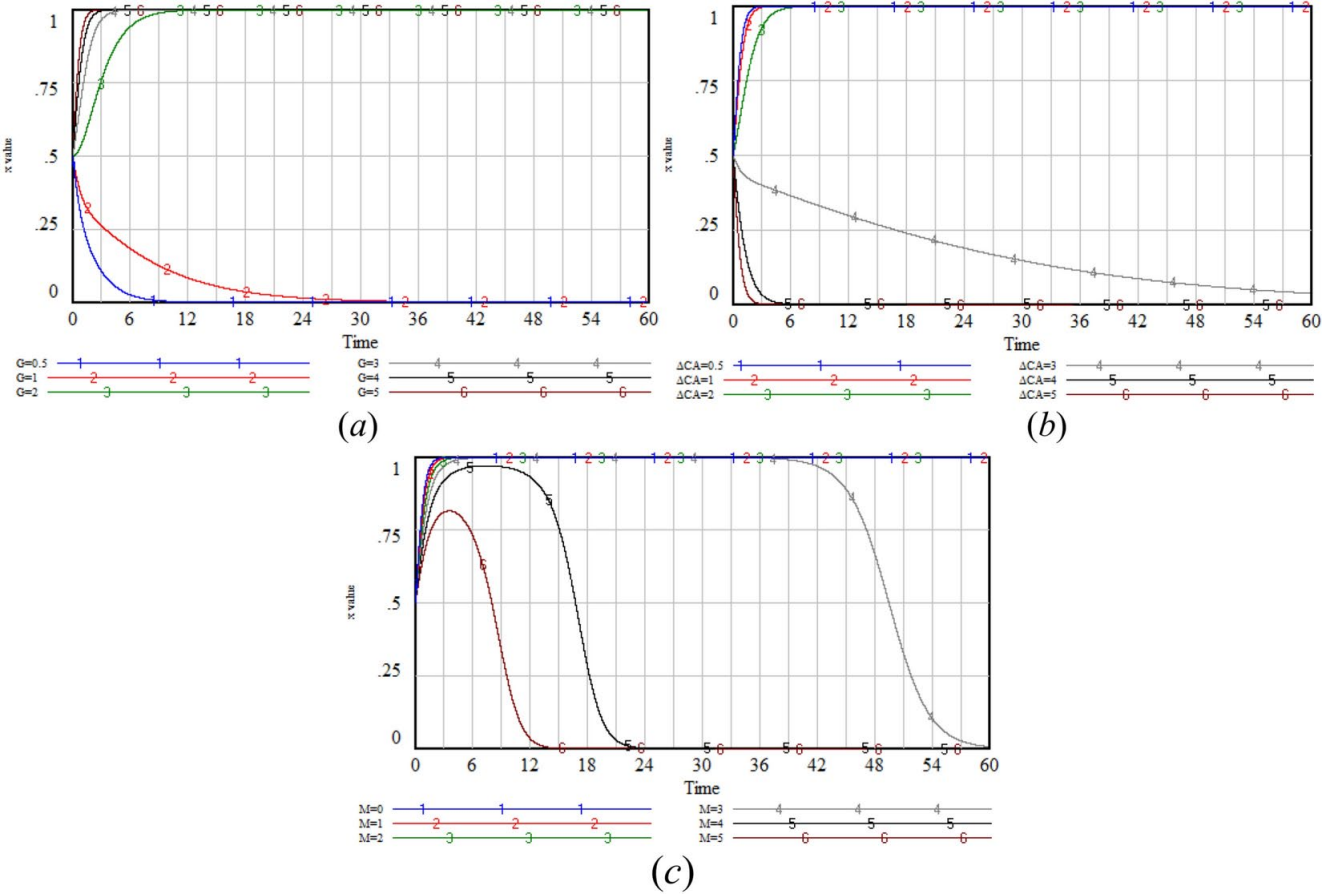
Sensitivity analysis of *x* under varying values of *G,* Δ*C*_*A*_* and M.*

The simulation results show that with the increase in *G* value, the *x* value increases significantly, which indicates that under the incentive of high reputation value, the government is more inclined to adopt strict regulatory strategy to maintain and enhance its positive image among the public. However, the increase of *M* value has a limited effect on *x* value. This may be because, when weighing reputation and costs, the government places greater value on long-term reputation accumulation than on short-term free-rider gains. When the Δ*C*_*A*_ value increases, the x value shows a downward trend, indicating that the cost factor imposes a constraint on the government’s decision-making. As the cost of regulation rises, the government may reassess the cost–benefit ratio of regulatory strategies, leading to a reduction in the preference for strict regulation (Fig. 7).

### GenAI company sensitivity analysis

With the aim of analyzing the impact of variables such as the benefits generated by optimization strategy (*R*_*BH*_), the additional cost of optimization (Δ*C*_*B*_), and the losses of company when risk events occur (*L*_*B*_) on the probability of the GenAI company adopting the optimization strategy (*y*) when facing privacy protection issues. We set six values ranging from small to large for each of *R*_*BH*_, Δ*C*_*B*_, and* L*_*B*_, while the other variables remain constant at initial values.

**Fig. 8:**
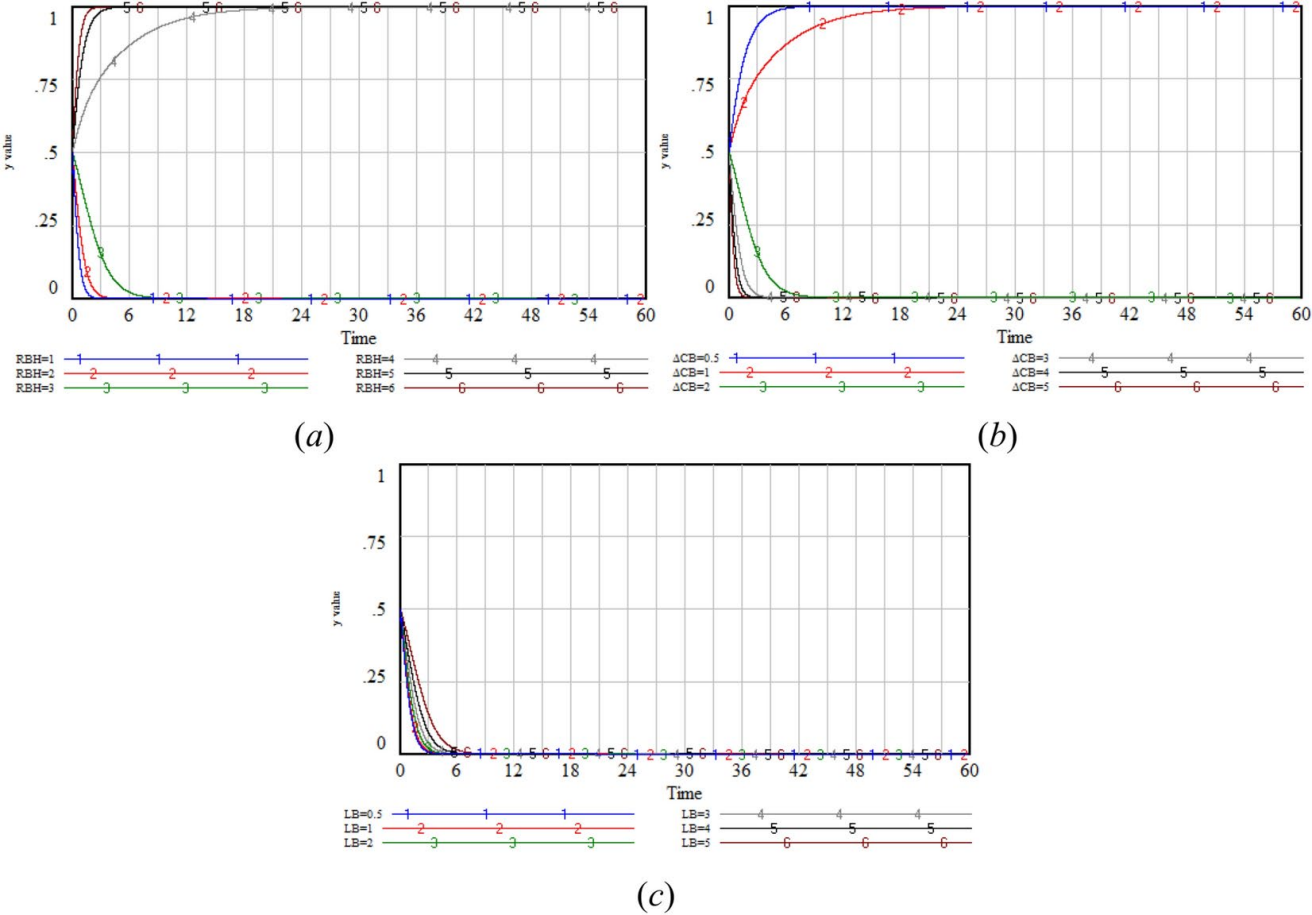
Sensitivity analysis of *y* under varying values of *R*_*BH*_*,* Δ*C*_*B*_* and L*_*B*_*.*

The simulation results show that with the increase in *R*_*BH*_ value, the *y* value increases significantly, which indicates that the GenAI company is very sensitive to the direct economic benefits and brand value improvement brought by optimization. Under the incentive of high returns, the GenAI company is more inclined to adopt the optimization strategy to maximize long-term interests. When the Δ*C*_*B*_ value increases, the *y* value shows a decreasing trend. This indicates that the additional cost input has a negative effect on the probability of the company choosing optimization. With the increase in costs, the GenAI company may re-evaluate the cost-effectiveness of optimization and reduce or postpone the optimization strategy. When *L*_*B*_ value increases, *y* value keeps evolving towards 0, which indicates that risk events are inevitable, and the loss of risk events is not enough to change the GenAI company’s strategy choice (Fig. 8).

### User sensitivity analysis

This section aims to analyze how key factors influence the probability of users choosing to disclose privacy to the GenAI company (*z*). These factors include the benefits generated by disclosure (*R*_*CH*_), additional cost of disclosure (Δ*C*_*C*_), and the losses of users when risk events occur (*L*_*C*_). We set six values ranging from small to large for each of *R*_*CH*_, Δ*C*_*C*_, and *L*_*C*_, while the other variables remain constant at initial values.

**Fig. 9:**
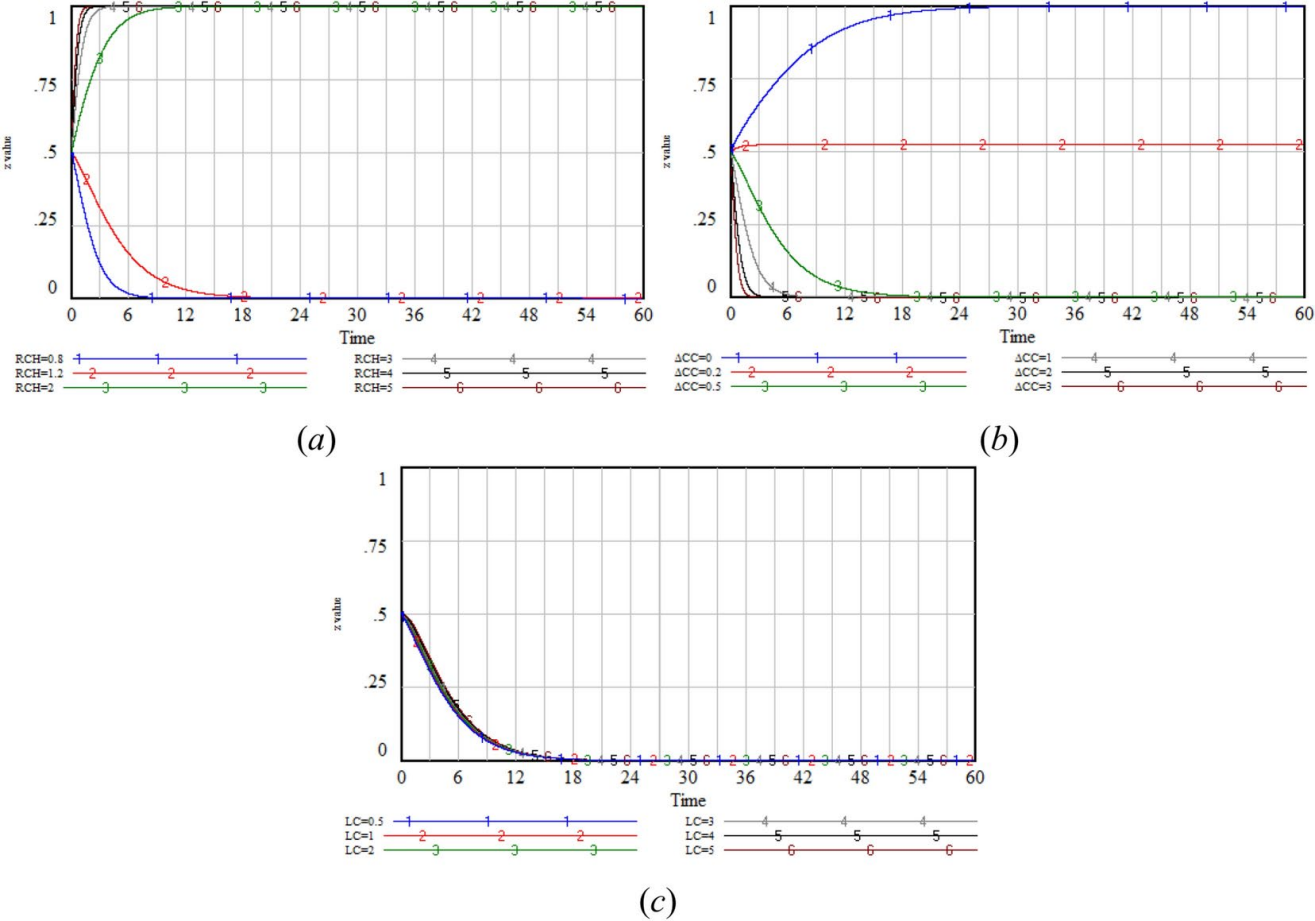
Sensitivity analysis of *z* under varying values of *R*_*CH*_, Δ*C*_*C*_, and *L*_*C*_*.*

The simulation results show that with the increase in *R*_*CH*_ value, the *z* value increases significantly, which indicates that under the incentive of high benefits, users are more willing to disclose privacy in exchange for rewards or services. When the Δ*C*_*C*_ value increases, the *z* value shows a decreasing trend. This indicates that with the increase of cost, users may reevaluate the cost-effectiveness of disclosure and reduce or even stop privacy disclosure. When the *L*_*C*_ decreases to a certain value, users will also choose not to disclose. This maybe because when using GenAI, users pay special attention to personal privacy and security, and are worried about the potential negative effects caused by the occurrence of risk events such as privacy breaches (Fig. 9).

### Incentive mechanism sensitivity analysis

Given that *F*_*AB*_ and *S* are only related to the government and the GenAI company, while *T* is only associated to the users. Therefore, the changes in the values of *F*_*AB*_ and *S* are analyzed to assess the sensitivity of *x* and *y*, the change in the value of *T* is analyzed to evaluate the sensitivity of *z*.

Incentive mechanism sensitivity analysis aims to examine how the key parameters in the incentive mechanism, namely fines (*F*_*AB*_), rewards (*S*), and integral rewards (*T*), affect the strategy choices of the government, GenAI company, and users in the three-party game.

**Fig. 10:**
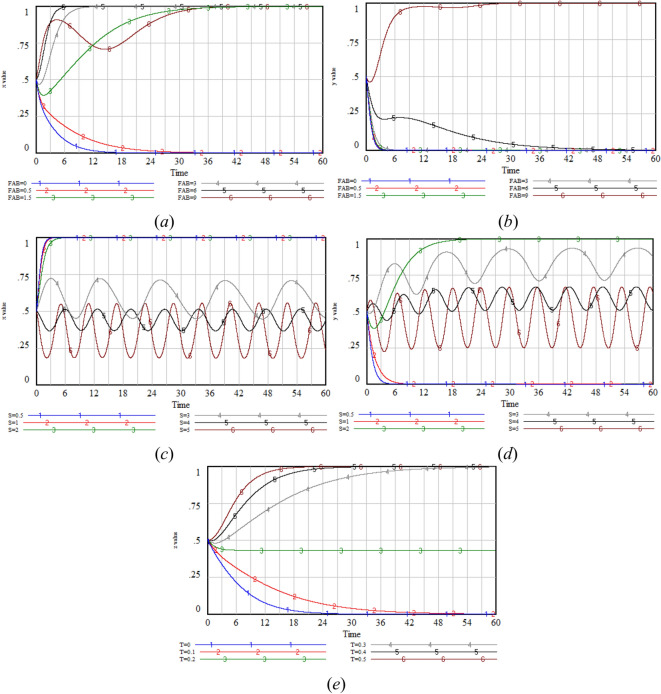
Sensitivity analysis of *x, y* under varying values of *F*_*AB*_, *S* and *T.*

The simulation results show that with the increase in *F*_*AB*_ value, the* x* value presents an upward trend, and when the *F*_*AB*_ value reaches a certain threshold, the *y* value increases significantly. This indicates that high fines are an effective measure to encourage GenAI company to adopt privacy protection measures. When *S* = 0.5 or *S* = 1, the government will choose strict regulation, but due to the low subsidy, the GenAI company chooses non-optimization. When *S* = 2, the government continues to choose strict regulation and the GenAI company chooses optimization, as the government’s reward effectively incentivizes the GenAI company to choose optimization. When *S* increases to more than 3, the decisions of the GenAI company and the government become intertwined with fluctuations. At this time, due to the excessively high subsidy, the government will tend to choose lax regulation. Under lax regulation, the government no longer provides subsidies to GenAI company. At this time, the GenAI company will also turn to choose non-optimization. As a result, there will be fluctuations between the GenAI company and the government. In reality, the government is unlikely to continue increasing subsidies once the GenAI company chooses optimization, which aligns with real-world practices. As the *T* value increases, the *z* value increases significantly. The integral reward directly motivates users to disclose privacy and enhances their willingness to participate (Fig. 10).

## Conclusion and practical suggestions

### Conclusions

The rapid advancement of generative artificial intelligence (GenAI) technology has presented both transformative opportunities and significant challenges to society. Users can utilize GenAI to accomplish a large number of tasks that satisfy their own needs. Meanwhile, GenAI company can benefit by providing these services. However, privacy breach risks exist when users interact with GenAI products. GenAI company can use the data disclosed by users to optimize privacy protection technology and reduce risks. But they must weigh the costs and benefits of such technological optimization. Currently, a key challenge in GenAI applications is how to motivate GenAI company to optimize technology for better user privacy protection. Consequently, a three-party game situation among the government, GenAI company, and users has emerged.

Therefore, this study constructs a three-party game model involving the government, GenAI company, and users. By adopting a combined approach of evolutionary game theory and system dynamics, it analyzes the interactive influences and evolutions of the three parties in terms of privacy protection strategies. Through simulation analysis, we conduct a sensitivity analysis of four types of factors, namely the government, GenAI company, users and incentive mechanisms, and reveal the impact of these factors on the strategic choices of the three parties. The research results show that the government’s regulatory decisions are deeply influenced by its emphasis on reputation, while the investment decisions of GenAI company and the information disclosure behaviors of users are significantly affected by the cost–benefit ratio and direct benefits respectively. The key findings highlight the importance of the intervention mechanisms in the strategic choices of the three parties. In particular, government subsidy and penalty policies play a promoting role in privacy protection, and subsidy policies are more effective than penalty policies.

This study not only provides a new perspective for understanding the privacy protection issues in human-intelligence interaction but also offers theoretical support and practical guidance for formulating effective privacy protection policies.

### Research limitations

Considering the potential differences between the model assumptions and realistic conditions, this study has several theoretical modeling limitations. First, the model simplifies governmental regulation to a binary choice between strict and lax regulation. whereas real-world implementations demonstrate multilevel dynamic adjustments. For example, the *EU AI Act* implements differentiated regulation according to the level of risk^45^. So the dynamic strategy adjustment based on evolutionary game provides a theoretical reference for such regulation^27^. In the future, a responsive regulatory framework can be introduced to enhance the explanatory power of model reality. Second, the static setting of enterprise optimization cost fails to reflect the dynamic coupling effect of technology iteration and risk events, so the dynamic cost mechanism of adaptive penalty strategy can be considered. Moreover, the fixed risk probability assumption fails to address probability drift caused by technological uncertainty. The future modeling can consider the dynamic evolution based on threshold switching. Third, the enterprise optimization preset in the model can significantly reduce the probability of risk events, but the lack of algorithm transparency in practice may weaken the actual effect of technical optimization^46^. It is necessary to further improve the risk modeling in combination with the technical characteristics of GenAI. Consequently, how to design effective privacy protection policies to balance the conflict between users’ privacy disclosure intentions and the efficiency of GenAI services remains a challenging issue.

### Practical suggestions

According to the research findings, several policy suggestions regarding privacy protection within the three-party game involving the government, generative AI company, and users have been put forward.

1. The government should continue to pay attention to reputation building. Given the rapid pace of technological and market change, adopting a rolling optimization strategy is necessary. This requires implementing a dynamic assessment system for regulatory effectiveness by incorporating core indicators such as regulatory cost Δ*C*_*A*_ and social benefit *R*_*BH*_ into real-time monitoring. The system should automatically trigger regulatory intensity adjustment procedures when Δ*C*_*A*_/*L*_*B*_ (regulatory cost-loss ratio) exceeds the threshold. Besides, government also needs to be wary of free-riding behavior, ensure the effectiveness and fairness of regulatory policies, and avoid damaging long-term reputation in pursuit of short-term gains. Moreover, the government can also consider adopting various strategies such as introducing cooperation with the private sector and using technological means to improve regulatory efficiency, in order to reduce regulatory costs and enhance regulatory effects.
2. GenAI company should continuously focus on the long-term economic and brand benefits brought by optimization. Meanwhile, they should reduce the cost of optimization through technological innovation and management optimization to enhance the cost-effectiveness of optimization. GenAI company should establish a dynamic cost–benefit evaluation system for technology optimization. This system would continuously monitor optimization cost (Δ*C*_*B*_), optimization income (*R*_*BH*_) and risk loss (*L*_*B*_) and other core indicators, when the risk loss to optimization cost ratio (*L*_*B*_/Δ*C*_*B*_) exceeds the industry threshold, the technology route optimization process will be automatically triggered. GenAI company also need to strengthen risk management and minimize potential risk losses through optimization, thereby improving their risk resistance capabilities. In response to government rewards (S) provided by the government, enterprises can design flexible and optimized investment strategies.
3. Users should continue to pay attention to the long-term economic and personalized service benefits brought by disclosure. At the same time, they should make wise decisions by reasonably evaluating costs and risks. Users also need to strengthen their awareness of personal privacy protection and reduce the risk of privacy breaches by adopting appropriate privacy protection measures. In addition, through the transparent privacy policies provided by government and strategies such as strengthening data security protection provided by GenAI company, users can reasonably disclosing personal information while protect their privacy rights and interests.
4. Government should consider implementing appropriate penalty policies and subsidy policies to effectively motivate GenAI company and users to take the expected actions. Although simulation results indicate that government subsidy policies have better effects than punishment policies, the amounts of fines and rewards need to be adjusted according to actual conditions to ensure the effectiveness and fairness of policies. It is recommended to implement a dynamic evaluation system for intervention mechanisms, focusing on monitoring the non-linear effects of government rewards (*S*) and government fine (*F*_*AB*_) on y (enterprise optimization probability) to improve the government’s accurate decision-making and dynamic adaptation capabilities. Meanwhile, GenAI company should realize a closed loop of user disclosure and optimization feedback, and analyze the correlation between disclosure probability (*z*), disclosure cost (Δ*C*_*C*_) and disclosure reward (*T*) in real time. When disclosure cost (Δ*C*_*C*_) lead user to reduce disclosure willingness, the user acceptance of technology optimization can be dynamically improved through reasonably design points-based reward mechanisms and transparent data use policies.

## Data Availability

The datasets used or analyzed during the current study are available from the corresponding author on reasonable request.
